# Loss of maturity and homeostatic functions in Tuberous Sclerosis Complex-derived astrocytes

**DOI:** 10.3389/fncel.2023.1284394

**Published:** 2023-11-28

**Authors:** Mark J. Luinenburg, Mirte Scheper, Frederik N. F. Sørensen, Jasper J. Anink, Wim Van Hecke, Irina Korshunova, Floor E. Jansen, Kate Riney, Pieter van Eijsden, Peter Gosselaar, James D. Mills, Rozemarijn S. Kalf, Till S. Zimmer, Diede W. M. Broekaart, Konstantin Khodosevich, Eleonora Aronica, Angelika Mühlebner

**Affiliations:** ^1^Amsterdam Neuroscience, Department of (Neuro)Pathology, Amsterdam UMC, University of Amsterdam, Amsterdam, Netherlands; ^2^Biotech Research and Innovation Centre (BRIC), Faculty of Health and Medical Sciences, University of Copenhagen, Copenhagen, Denmark; ^3^ERN EpiCare, Department of Pathology, Brain Center, University Medical Center, Utrecht, Netherlands; ^4^ERN EpiCare, Department of Child Neurology, Brain Center, University Medical Center, Utrecht, Netherlands; ^5^Faculty of Medicine, The University of Queensland, Herston, QLD, Australia; ^6^Neurosciences Unit, Queensland Children’s Hospital, South Brisbane, QLD, Australia; ^7^Department of Neurosurgery, University Medical Center, Utrecht, Netherlands; ^8^UCL Queen Square Institute of Neurology, London, United Kingdom; ^9^Chalfont Centre for Epilepsy, Buckinghamshire, United Kingdom; ^10^Stichting Epilepsie Instellingen Nederland (SEIN), Heemstede, Netherlands

**Keywords:** astrocytes, inflammation, phagocytosis, glutamate buffering, TSC, epilepsy

## Abstract

**Introduction:**

Constitutive activation of the mTOR pathway, as observed in Tuberous Sclerosis Complex (TSC), leads to glial dysfunction and subsequent epileptogenesis. Although astrocytes are considered important mediators for synaptic clearance and phagocytosis, little is known on how astrocytes contribute to the epileptogenic network.

**Methods:**

We employed singlenuclei RNA sequencing and a hybrid fetal calf serum (FCS)/FCS-free cell culture model to explore the capacity of TSC-derived astrocytes to maintain glutamate homeostasis and clear debris in their environment.

**Results:**

We found that TSC astrocytes show reduced maturity on RNA and protein level as well as the inability to clear excess glutamate through the loss of both enzymes and transporters complementary to a reduction of phagocytic capabilities.

**Discussion:**

Our study provides evidence of mechanistic alterations in TSC astrocytes, underscoring the significant impairment of their supportive functions. These insights enhance our understanding of TSC pathophysiology and hold potential implications for future therapeutic interventions.

## Graphical Abstract

**(A)** Changes in homeostatic functions between control and TSC astrocytes. **(B)** Workflow used in this study.

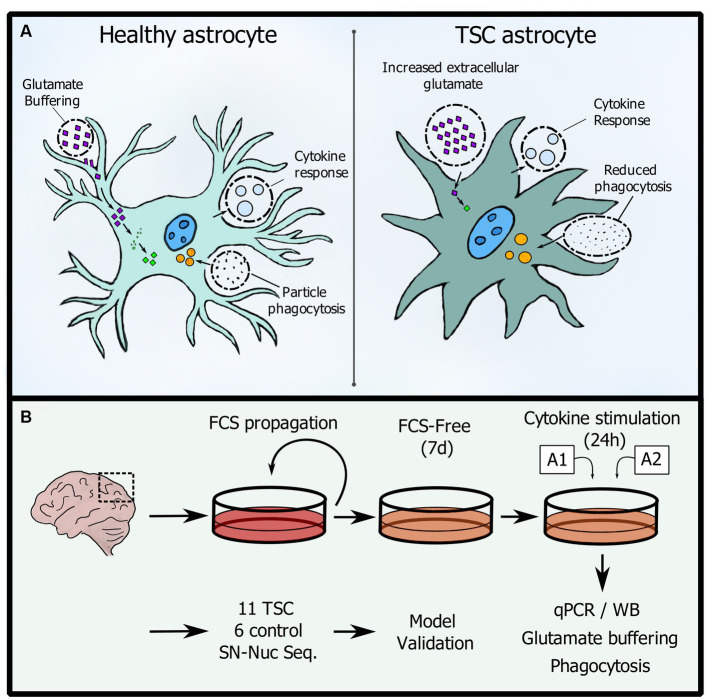

## Introduction

The term ‘astrocyte reactivity’ has been introduced as being broadly equivalent to reactive astrogliosis, but emphasizing the capacity of astrocytes to adopt distinct state(s) in response to diverse pathologies (Escartin et al., 2019; Wheeler et al., 2020). Therefore, ‘reactive astrocytes,’ referring to the cells undergoing this remodeling, is an umbrella term encompassing multiple potential states. ‘State’ is defined as a transient or long-lasting astrocyte condition characterized by a specific molecular profile, specific functions, and distinct impact on diseases, while ‘phenotype’ is the measurable outcome of that state (Sofroniew, 2015). Importantly, the changes in astrocytes in response to pathological stimuli are not to be confused with the plasticity of healthy astrocytes, which are constantly being activated by physiological signals in the central nervous system (Ben Haim and Rowitch, 2017). Hence, although the transitions from a physiological to a pathological state are progressive and occasionally challenging to define, the term ‘astrocyte activation’ should be reserved for physiological conditions. In pathological contexts, it should be referred to as ‘astrocyte reactivity’ (Escartin et al., 2019). Astrocytes display distinct functional changes in a variety of epilepsies with different etiologies and many studies have suggested that they play crucial roles in the process of epileptogenesis, including Tuberous Sclerosis Complex (TSC) (Binder, 2018; Binder and Steinhäuser, 2021). TSC is a genetic disorder characterized by the loss of function of either *TSC1* or *TSC2* genes, resulting in the hyperactivation of the mechanistic target of rapamycin (mTOR) pathway at the molecular level. mTOR dysregulation in the brain leads to altered cortical development, resulting in the formation of focal lesions known as tubers, which are associated with a wide range of neurological manifestations, including epilepsy (reviewed in Curatolo et al., 2022; Aronica et al., 2023). Neuropathological hallmarks in resected cortical tubers of TSC patients include morphologic and functional changes in astrocytes. While most studies characterize the total population of astrocytes, some report different subpopulations of astrocytes in TSC (Talos et al., 2008). Previous studies characterized two subpopulations of astrocytes: ‘reactive’ cells, which are large and vimentin positive and reveal mTOR activation, and ‘gliotic’ astrocytes, which are smaller, do not show mTOR activation, and resemble gliotic astrocytes found in temporal lobe epilepsy with hippocampal sclerosis (TLE-HS) (Sosunov et al., 2008, 2012). Additional data supports the notion that populations of improperly differentiated astrocytes with mTOR activation, in combination with properly developed reactive astrocytes without mTOR activation, contribute to TSC pathology (reviewed in Zimmer et al., 2020). Importantly, the functional changes in TSC astrocytes are likely caused by a combination of the reactive state in response to seizures which could induce secondary mTOR activation (Sosunov et al., 2012), but also general disturbance in protein translation caused by sustained mTOR activation in mutation-carrying cells (Zimmer et al., 2020). Determining whether the varying degrees of mTOR activation underlie the wide diversity of astrocyte functions and phenotypes in TSC requires further investigation (Zimmer et al., 2020). Nevertheless, it has already been demonstrated for neurons that the extent of mTOR hyperactivity correlates with the severity of seizures and associated neuropathology (Nguyen et al., 2019). Finally, in addition to intrinsic astrocytic properties, maintenance of a non-reactive state in astrocytes was also shown to depend on neuronal mTORC1 activity, adding yet another dimension in which astrocyte function in TSC is altered (Zhang et al., 2017).

Herein, we hypothesize that TSC astrocytes are impaired due to their response patterns being trapped in an immature phenotype. Therefore, we have evaluated astrocytic reaction patterns to inflammatory stimulation and their capacity to maintain glutamate levels and engage in phagocytosis (see Graphical Abstract).

## Methods

### RNA isolation

RNA was isolated using Trizol (Invitrogen). In short, cells were washed with phosphate buffered saline (PBS), lysed, and RNA extracted using phenol-chloroform extraction (1:0.14). These were separated by centrifugation (12,000 × g, 15 min, 4°C). The aqueous phase was retrieved and mixed with equal volume isopropanol and 1 μL glycogen-blue (Invitrogen). Samples were precipitated overnight at −20°C. Following day, RNA was pelleted by centrifugation (>20,000 × *g*, 30 min, 4°C) and washed twice with ice-cold 75% ethanol. Pellets were then air dried for 5′ at RT, dissolved in THE RNA solution (Invitrogen) with an additional 20 mM DTT and heated to 60°C for 10′ to deactivate residual RNases.

### cDNA synthesis and qPCR

Isolated RNA was quantified using a Nanodrop spectrophotometer (ThermoFisher Scientific) and 250 ng/reaction was used for cDNA synthesis using oligo-DT primers. Quantitative qPCR was performed with a Lightcycler 480 (Roche Applied Science) with the small nuclear ribonucleoprotein D3 polypeptide (SNRPD3) as reference gene. Quantification was performed using the LinRegPCR method as described previously (van Scheppingen et al., 2016). Outliers were removed using a ROUT algorithm with a *Q* = 0.5% and visualized in Graphad (8.4.3, GraphPad Software Inc). Mann–Whitney *U* tests were performed to test for significance between groups.

### Cell isolation and culture

Cells were isolated from surgically resected tubers from TSC patients (aged 3 to 26 years; *TSC 1/2* mutation, median 10.8y, 60% Male: 40% Female, see Supplementary Table S1 for detailed patient information) using a papain dissection method (Worthington). For control astrocytes, cells were isolated from gray/white matter frontal-cortical human fetal brain tissue derived from abortions (without genetic indication). Tissue was collected with written consent and according to the declaration of Helsinki as well as the Amsterdam research code of the medical ethics committee. In detail, brain tissue was collected in 1:1 Dulbecco’s modified eagle medium/F12 (DMEM/F12) + 10% fetal calf serum (FCS) + 100 μg/mL penicillin, 100 μg/mL streptomycin or Hibernate-A (Gibco) media. Before the dissociation, the tissue was washed and collected into 13 mm plates containing Dulbecco’s phosphate buffered saline (dPBS) + 10 μM Y-27632. Tissue was cut into <1 mm^3^ segments, collected, left to settle and excess buffer was removed. The cell suspension was mixed with papain solution (1x Hanks’s balanced salt solution, 0.46% glucose, 25 mM HEPES pH 7.5, 1.7 mM L-cysteine, 10 μM Y-27632) with a concentration of 7.5 units/mL papain (Worthington) for fetal and 20 units/mL for TSC tissue. The tissue suspension was then incubated for 40–60 min at 34°C with intermittent mixing. The protease was deactivated with inhibitor solution (DMEM/F12, 1% FCS, 0.0005% DNase) and gently broken up by repeated pipetting through a serological pipet, moving the supernatant with single cells into a fresh tube. Cells were spun down and suspended in astrocyte maintenance media (DMEM/F10 1:1, 10% FCS, 1 mM glutamine, 100 μg/mL penicillin, 100 μg/mL streptomycin). This pooled isolate was filtered with a cell strainer (70 μM) and distributed into flasks. All experiments were performed with cells passage 3–7. Experiments were performed after 7 days in FCS-free formulations (see below). Cells were screened visually and for CD45 mRNA as indicator of microglia presence.

### Cytokine stimulation assays

Cells were stimulated using the previously established A1, A2 combinations (Liddelow et al., 2017). Briefly, the cells were seeded at 1.25 × 10^4^/cm2 on 0.25% Matrigel and cultured in FCS-free media containing DMEM/F12 + 1x N2, 0.1x B27, 20 μM n-acetylcysteine, 1 mM Glutamax, 5 ng/mL fibroblast growth factor 2 (FGF2), 8 ng/mL bone morphogenetic protein 4 (BMP4), 5 ng/mL ciliary neurotrophic factor (CNTF), 2.5 ng/mL heparin binding EGF like growth factor (HBEGF). After 7 days the media was changed to non-treated (NT) consisting of DMEM/F12, 1x N2, 0.1x B27 supplemented with an additional A1 (30 ng tumor necrosis factor alpha - TNF-α, 3 ng interleukin 1 alpha - IL-1α, 400 ng complement component 1q – C1q/mL) or A2 (30 ng TNF-α, 10 ng interleukin 1 beta - IL-1β/mL) mix for stimulated conditions. After 24 h cells were washed with PBS and lysed for RNA and protein. For glutamate and phagocytosis assays the DMEM/F12 was replaced by BrainPhys (StemCell, #05790) media whilst keeping all additives (N2, B27 etc.) consistent. Refer to Supplementary Table S3 for details on the reagents used.

### Glutamate clearance assay

Supernatant was diluted 60x and measured by Glutamine/Glutamate-Glo Assay kit (Promega) according to the manufacturer’s instructions in 384 well format. For normalization to cell viability, we used calcein (Biolegend) at 1 μM according to the manufacturer’s instructions. Fluorescent intensity was measured with a Clariostar plate reader (BMG Labtech).

### Protein isolation and immunoblotting

Cells were lysed for 30 min at 4°C using radio immunoprecipitation assay buffer (RIPA) buffer supplemented with 2x Protease Inhibitor Cocktail Set Vl (Merck) and 1x PhosSTOP (Roche). Samples were quantified and blotted as described earlier (Gruber et al., 2022). For primary antibodies, secondary antibodies and reagents overview see Supplementary Table S2. ECL solution (Pierce ECL Western Blotting Substrate, Thermofisher) or enhanced ECL solution (SuperSignal West Pico PLUS Chemiluminescent Substrate, Thermofisher) was used for development of membranes. When necessary, membranes were stripped with Restore PLUS Western Blot Stripping Buffer (Thermofisher) according to the manufacturer’s instructions, blocked and reprobed. For a detailed description see Supplementary Methods.

### Crude synaptic fraction isolation and tagging

Fetal brains (gestational week 15–24) were processed using the Syn-PER Synaptic Protein Extraction Reagent (ThermoFisher) protocol with modifications: samples were washed an additional time with isolation reagent before storage to increase purity (Carlyle et al., 2021) and isolations were frozen down in storage buffer (Nath et al., 2014) (0.34 M Sucrose, 10% DMSO, 2 mM EDTA, 5 mM Tris pH 7.4). BCA was performed on DMSO and EDTA free aliquots of crude synaptosomes and enrichment was confirmed by western blot analysis of synaptophysin in whole, cytosolic and crude synaptic fractions (see Supplementary Figure S1). For pHrodo red conjugation, samples were thawed at 37°C, spun down (16,000 × g, 5′) and resuspended in 500 μL 0.1 M bicarbonate buffer pH 9.0 to an final concentration of 2 mg/mL. pHrodo Red (Thermofisher) was added with a 125 μM /mg ratio. Reactions were incubated for 1 h in the dark at RT, washed with PBS, resuspended in dPBS –Mg^2+^ –Ca^2+^ (Gibco) to approximately 2 mg/mL, necessary analysis aliquots were made and the samples were further diluted 1:1 with dPBS –Mg^2+^ –Ca^2+^ + 10% DMSO to yield a concentration of 1 mg/mL. Ampules were frozen at −80°C in a controlled manner and transferred to liquid nitrogen (LN_2_). For verification of labeling, 10x diluted samples were subjected to a pH titration curve (pH 2; 7; 10) and fluorescent intensity was measured with a Clariostar plate reader (BMG Labtech, Ex 540–20, Em 590–30) followed by protein determination with a Nanodrop spectrometer (Thermofisher, 280 nm).

### Phagocytosis assay

Cells were cultured as described above and cultured in 96 well black plates (PhenoPlate, Perkin Elmer). After 24-h stimulation with A1 or A2, 2.5 μg of pHrodo labeled crude synaptic fraction were added per well. For mTOR inhibition 100 nM rapamycin treatment (Selleck Chemicals) was used for 24 h as described previously (Broekaart et al., 2017). Cells were measured hourly for 24 h in an Incucyte S3 (Sartorius) in technical duplicates with 4 images per well. For data analysis, the mean red signal was baseline subtracted. Mann–Whitney *U* tests were performed to test for significance between groups.

### Cohort snRNA-seq

Brain tissues included in this study were obtained from the archives of the Departments of Neuropathology of the Amsterdam UMC (Amsterdam, The Netherlands), the UMC Utrecht (Utrecht, The Netherlands) and Queensland Children’s Hospital (Brisbane, Australia). Frontal cortex tissue was flash frozen in LN_2_ upon resection and control samples were age and location matched. Six control samples and 11 TSC samples were sequenced using snRNA-seq methods with RNA integrity number > 5. Selected cohort metrics can be found in Supplementary Table S1.

### Nuclei extraction and FACS sorting

Nuclei extraction and FACs sorting was performed as described in detail before (Krishnaswami et al., 2016; Pfisterer et al., 2020; Batiuk et al., 2022). The tissue samples, including both TSC and control samples, were processed in parallel whenever possible. Tissue was removed from −80°C and transferred to chilled homogenization buffer and homogenized. The resulting homogenate was filtered through a 40-μm cell strainer and centrifuged at 1,000 *g* for 8 min at 4°C. Supernatant was removed and the pellet was resuspended in 250 μL 0.5% bovine serum albumin (BSA) in 1X PBS with RNAse inhibitor (Takara, 2313B, final concentration 0.4 U/μl) for blocking and was incubated for 15 min on ice. Subsequently, samples were stained with anti-NeuN antibody Ms-NeuN-488 (Millipore, MAB3777x, 1 μg/μL, 1:1890) and incubated in the dark for 10 min at 4°C. Afterwards, the suspensions were centrifuged at 1,000 *g* for 8 min at 4°C, and the pellets were resuspended and filtered through 35 μM strainers into fluorescence-activated cell sorting (FACS) tubes resulting in a final volume of 500 μL. To gate for nuclei 0.75 μL of 7-aminoactinomycin (7-AAD), a nucleic acid chelating fluorophore, was added to samples on ice. Immediately after, FACS was performed and NeuN positive cells were enriched and sorted into BSA pre-coated 1.5 mL Lo bind Eppendorf tubes at 4°C, 20% of negative NeuN fraction was added to the enriched fraction to yield the final sample composition (80% NeuN+, 20% NeuN- nuclei).

### Library preparation and sequencing

RNA-sequencing library preparation and sequencing were also performed as described in detail before (Pfisterer et al., 2020; Batiuk et al., 2022). The Chromium Single Cell 3’ Reagent Kits v3.1 from 10x Genomics were employed for library preparation. The procedure involved counting the nuclei under a microscope and combining them with reverse transcription mix and v3.1 Gel Beads on Chromium Chip G. This mixture was partitioned into Gel Beads-in-emulsion (GEMs) using the Chromium Controller. Following reverse transcription, the samples were frozen for up to a week. Afterwards, up to four samples from different 10x runs were processed together for cDNA cleanup and preamplification. The cDNA was then quantified on the Qubit HS dsDNA Assay Kit (Thermo Fisher Scientific, Q32854), Qubit Fluorometer and High Sensitivity DNA Kit (Agilent, 5,067–4,626) and Agilent 2,100 Bioanalyzer and the same quantity was used for fragmentation, end-repair, and A-tailing. Fragments were cleaned up, and subsequent steps included adapter ligation, cleanup, and sample index PCR. The libraries were cleaned up, quantified using the Agilent 2,100 Bioanalyzer system, and pooled based on the expected number of nuclei per sample. Finally, the libraries were sequenced on two 100 cycle NovaSeq 6,000 S2 flow cells (Illumina, 20012861) using a Illumina NovaSeq 6,000 (Illumina, 20012850).

### Unsupervised clustering of snRNA-seq data

After pre-processing, including CellBender 0.2.2 and filtering of the data, Seurat (v.4.1.3) was used to further process the data, following the guidelines for snRNA-seq data (Hao et al., 2021). For each sample, an expression matrix containing Unique Molecular Identifiers (UMIs) per nucleus per gene was imported as a 10x data object. Only nuclei with more than 200 genes and less than 5% of genes origination from mitochondrial sources were retained. Data was then imported as a Seurat object and all samples were integrated using the FindIntegrationAnchors and IntegrateData functions. The count matrix was scaled and normalized by variance stabilizing transformation (VST) with Seurat’s ScaleData and NormalizeData commands, respectively. The 2,000 most variable features were then selected with the FindVariableFeatures command for the Principal Component Analyses (PCA), which was performed by the RunPCA command. The PCs generated by the PCA were assessed with ElbowPlot and JackStraw analyses by using up to 20 different components. The resulting PCs were used for Jaccard-weighted, shared nearest neighbor (SNN) distance calculations and graph generation. The graph was then subjected to Louvain clustering and Uniform Manifold Approximation and Projection (UMAP) for dimension reduction in order to visualize nuclear transcriptomic profiles in two-dimensional space. After changing the default assay of the dataset from integrated to RNA, GFAP, ALDH1L1, AQP4, SLC1A2 and S100B were used to identify the astrocyte cluster. Further analyses were performed on this identified astrocyte group.

### Pseudo-bulk differential expression and enrichment analysis

To perform differential expression analysis between control and TSC samples, we performed pseudo-bulk analysis. This approach involves aggregating cells within each biological sample to create ‘pseudobulks.’ This aggregation is essential because single cells within the same biological sample are not independent of each other. Specifically, we aggregated astrocyte counts for each sample and generated a corresponding metadata column. Differential expression analysis was performed using the R package DESeq2 (Love et al., 2014). To control the false discovery rate, we applied the Benjamini–Hochberg correction, considering gene expression changes with an adjusted value of *p* < 0.05 as statistically significant. Subsequently, we visualized differentially expressed genes through volcano plots and conducted Gene Ontology (GO) enrichment analysis using the R package ‘clusterProfiler’. For this analysis, we adopted a cutoff criterion of an adjusted value of *p* < 0.05.

## Results

### TSC astrocytes are locked in an immature phenotype

**Figure 1:**
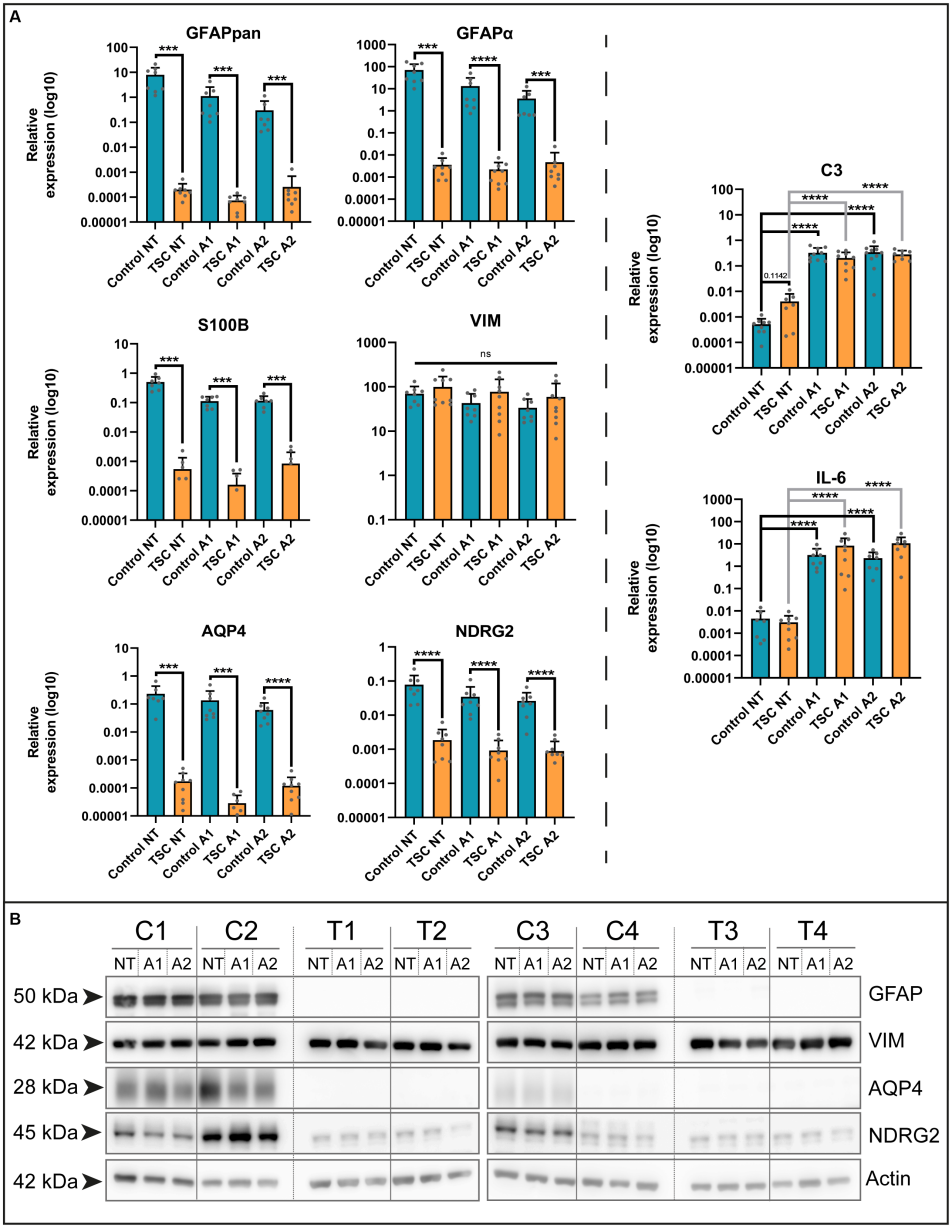
TSC astrocytes are locked in an immature phenotype. **(A)** Various markers of mature astrocytes (*GFAPpan, GFAPα, NDRG2, S100B*, and *AQP4*) were altered in TSC astrocyte cultures compared to control on mRNA level. In contrast, reactivity (*C3*, *IL-6*) had no significant differences in their baseline or reaction to stimuli. Data were normalized on *SNRPD3* housekeeping gene and plotted as relative expression. **(B)** Confirmation of various maturity markers on protein level. Data was derived from 5 TSC lines and 4 control lines from two separate experiments. **** indicates *p* < 0.0001, *** *p* < 0.0001, ** *p* < 0.001, * *p* < 0.05. NT: not treated; A1: C1q, TNF-α, IL-1α treated; A2: TNF-α, IL-1β treated. Mann–Whitney *U* tests without multiple test correction were performed to determine significance. *GFAPpan* (WT vs. TSC; NT: Log2 FC –15.26, *p* = 0.0002; A1: –13.88, *p* = 0.0001; A2: –10.20, *p* = 0.0003), *GFAPα* (WT vs. TSC; NT: Log2 FC –14.26, *p* < 0.0001; A1: –12.53, *p* < 0.0001; A2: –9.57, *p* < 0.0001), *NDRG2* (WT vs. TSC; NT: Log2 FC –5.40, *p* < 0.0001; A1: –5.12, *p* < 0.0001; A2: –4.88, *p* < 0.0001), *S100B* (WT vs. TSC; NT: Log2 FC –9.84, *p* = 0.0003; A1: –9.46, *p* = 0.0002; A2: –9.46, *p* = 0.0002), *AQP4* (WT vs. TSC; NT: Log2 FC –10.39, *p* = 0.0002; A1: –12.21, *p* = 0.0002; A2: –9.01, *p* = 0.0001).

To establish our model, we isolated human astrocytes in a hybrid FCS/FCS-free culture system (see Graphical Abstract for workflow). In this system, cells were initially expanded under traditional FCS conditions and were subsequently transferred to a media containing a mixture of maturing cytokines and FCS-free growth supplements for 7 days to induce a mature resting state (data not shown). Afterwards, cells were exposed to stimuli to induce neurotoxic (A1) or neuroprotective (A2) reactivity (Liddelow et al., 2017) (A1: C1q, TNF-α, IL-1α; A2: TNF-α, IL-1β). Using this paradigm, we conducted qPCR analysis on control and TSC astrocytes to identify differences in cellular responses that might be of interest. First, we examined a selection of genes based on commonly used markers of astrocytes in the central nervous system (CNS). These included: NDRG Family Member 2 (*NDRG2*, mature non-reactive) (Flügge et al., 2014), calcium-binding protein B (*S100B*, maturity) (Raponi et al., 2007), Aquaporin-4 (*AQP4*, maturity) (Ciappelloni et al., 2019), Glial fibrillary acidic protein (*GFAP*, reactivity/maturity) (Eng, 1985; Eng and Ghirnikar, 1994) and Vimentin (*VIM*, reactivity/maturity) (Pixley and de Vellis, 1984). Our approach revealed a decrease in the expression of maturity markers, including *NDRG2*, *S100B*, *AQP4*, and both *GFAPpan* and its astrocyte-specific isoform *GFAPα* (Roelofs et al., 2005) in TSC cultures (Figure 1A and Supplementary Table S3). Immaturity marker *VIM* was not differentially expressed between control or TSC astrocytes. To determine if differences in maturity influence the inflammatory response, we also investigated the expression of pro-inflammatory markers *C3* and *IL-6*. Both showed similar reaction to stimulation cues between control and TSC (Figure 1A), thus changes cannot be attributed to a higher inflammatory response. We validated our gene expression levels with western blot analysis on a subset of genes of interest (Figure 1B). Overall, TSC astrocytes showed a remarkable decrease of astrocytic markers associated with mature phenotypes but no changes in immature marker VIM or C3/Il-6 reactivity.

### Extracellular glutamate levels are improperly maintained by TSC astrocytes

**Figure 2:**
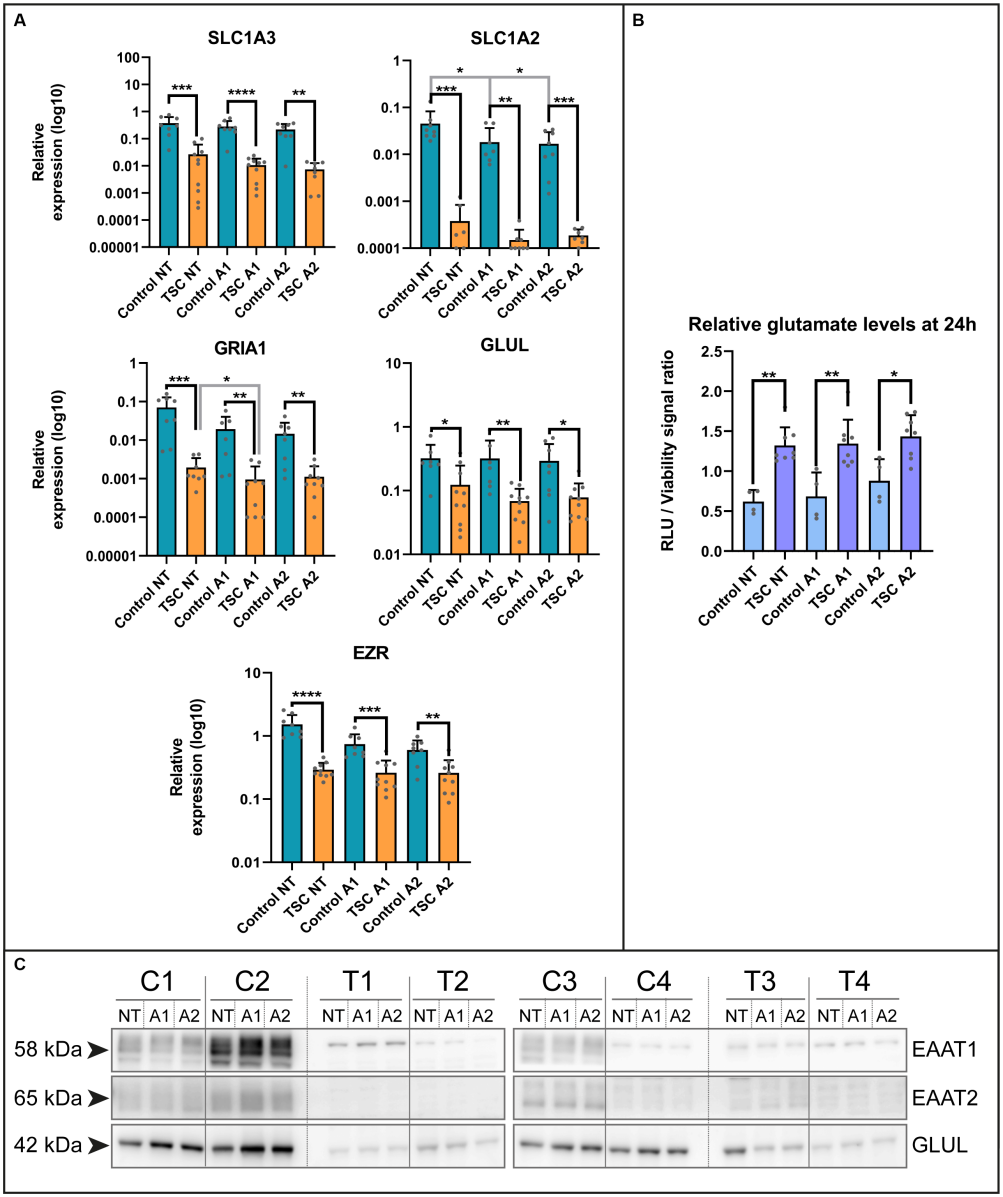
Extracellular glutamate levels are improperly maintained by TSC astrocytes. **(A)** mRNA levels of glutamate transporters (*SLC1A3, SLC1A2*), glutamine ligase (*GLUL*), glutamate receptors (*GRIA1*) and adapter protein (*EZR*) in control and TSC astrocytes using qPCR (5 TSC, 5 Control from two separate experiments). Data were normalized on *SNRPD3* housekeeping gene and plotted as relative expression. **(B)** Relative levels of glutamate showed impaired glutamate homeostasis in TSC astrocytes. TSC astrocytes had a significantly higher extracellular end concentration post-incubation compared to control. (4 TSC, 2 control in technical duplicates from two separate experiments). **(C)** Western blot analysis of SLC1A3, SLC1A2 and GLUL in 4 controls and 4 TSC primary cell lysates under (un) stimulated conditions. **** indicates *p* < 0.0001, ***p < 0.0001, **p < 0.001, *p < 0.05. RLU: relative light units, NT: not treated; A1: C1q, TNF-α, IL-1α treated; A2: TNF-α, IL-1β treated. Mann–Whitney *U* tests without multiple test correction were performed to determine significance. *SLC1A3* (WT vs. TSC; NT: Log2 FC –3.82, *p* = 0.0002; A1: –4.73, *p* < 0.0001; A2: –4.89, *p* = 0.0011), *SLC1A2* (WT vs. TSC; NT: Log2 FC –6.90, *p* = 0.0003; A1: –6.94, *p* = 0.0095; A2: –6.49, *p* = 0.0003), *GLUL* (WT vs. TSC; NT: Log2 FC –1.39, *p* = 0.0250; A1: –2.23, *p* = 0.0012; A2: –1.91, *p* = 0.0434), *GRIA1* (WT vs. TSC; NT: Log2 FC –5.19, *p* = 0.0002; A1: –4.33, *p* = 0.0021; A2: –3.70, *p* = 0.0025), *EZR* (WT vs. TSC; NT: Log2 FC –2.38, *p* < 0.0001; A1: –1.50, *p* = 0.0005; A2: –1.21, *p* = 0.0044).

Based on the lack of maturity, we explored potential alterations in key functions performed by astrocytes within the neuronal environment, including glutamate buffering (Mahmoud et al., 2019). Using qPCR, we examined various genes of interest in our *in vitro* system under two different cytokine stimulations, following a previous study on astrocyte reactivity (Liddelow et al., 2017). The selected genes were associated with the glutamate system, such as transporters (Excitatory amino acid transporter 1, *EAAT1* /solute carrier family 1 member 3 *SLC1A3*; Excitatory amino acid transporter 2, *EAAT2* /solute carrier family 1 member 3, *SLC1A2*), converting enzyme (glutamine synthetase, *GS* /Glutamate-Ammonia Ligase, *GLUL*), glutamate receptor subunit (Glutamate Ionotropic Receptor AMPA Type Subunit 1, *GRIA1*) and their associated protein Ezrin (*EZR*; Figure 2A and see Supplementary Table S3 for statistical details). All the genes of interest exhibited downregulation in TSC astrocytes and a selection of these targets was confirmed at protein level by western blot analysis (Figure 2C). Among the genes analyzed, we observed downregulation at the mRNA level for all investigated genes, as well as reduced protein levels for SLC1A3, SLC1A2, and GLUL.

Subsequently, we assessed whether the dysregulation of glutamate-related genes translated into functional deficiencies in homeostasis. We conducted a 24-h incubation with 1 mM glutamate in the presence or absence of cytokines (24 h pre-exposure), the remaining glutamate in the supernatant was measured and normalized with the cell viability dye calcein. With this approach, we could observe a significant increase in the glutamate levels present compared to control (WT vs. TSC; Log2 FC NT: 1.09, *p* = 0.004; A1: 0.97, *p* = 0.004; A2: 0.70, *p* = 0.0283; corrected for cell viability, see Figure 2B). This was in agreement with the non-normalized glutamate signal (data not shown). Based on this assay, TSC astrocytes maintained significantly higher extracellular glutamate levels, irrespective of inflammatory conditions.

### Phagocytosis of crude synaptic particles is severely impaired in TSC astrocytes

**Figure 3:**
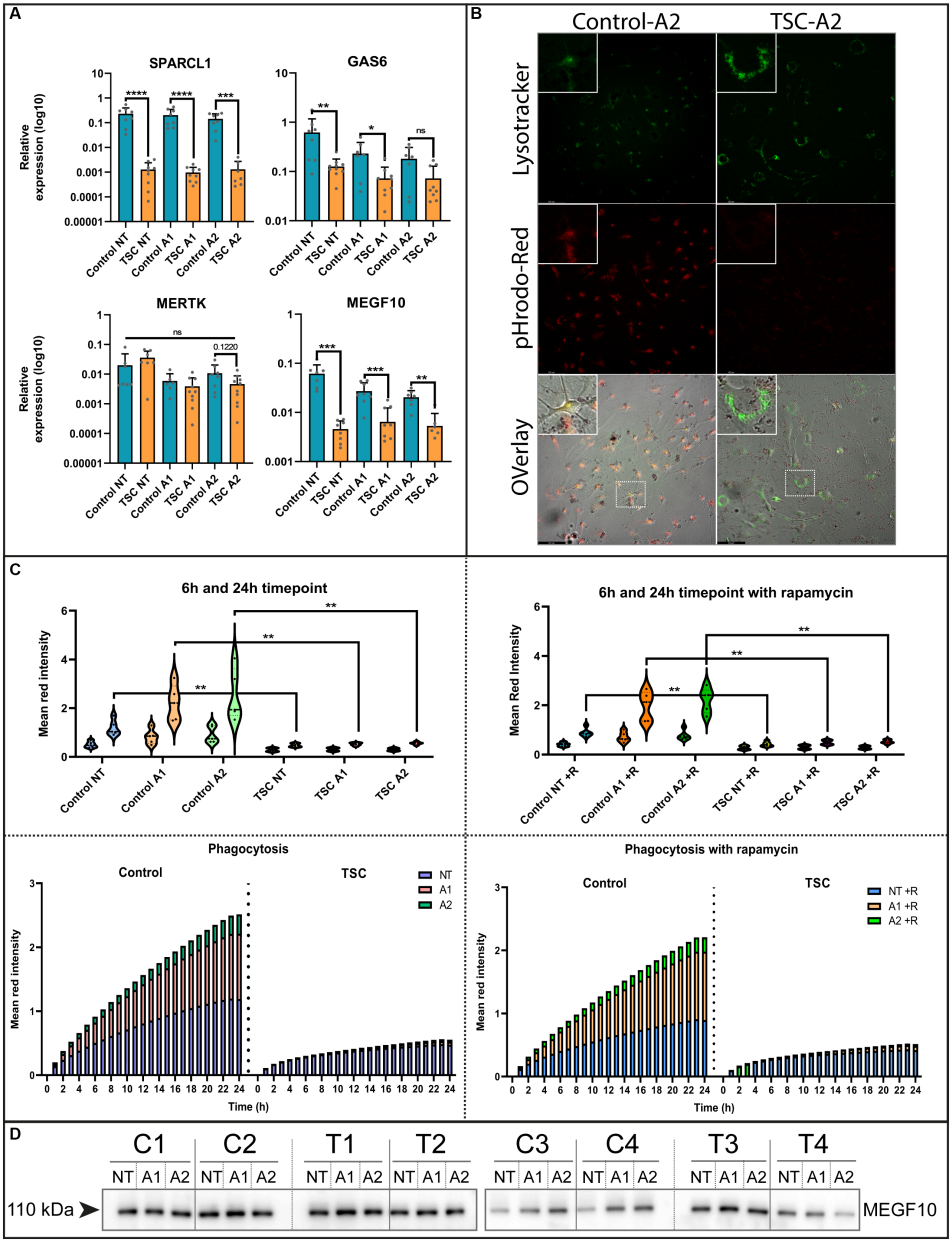
Phagocytosis of crude synaptic particles is severely impaired in TSC astrocytes. **(A)** qPCR of known phagocytotic (*MERTK*, *MEGF10*, *GAS6*) or synaptosomal interacting protein (*SPARCL1*) (5 TSC, 5 Control from two separate experiments). Data were normalized on *SNRPD3* housekeeping gene and plotted as relative expression. **(B)** Example of phagocytosis in a stimulated sample using a Lysotracker green – pHrodo-red imaging (24 post addition). **(C)** Clearance of pHrodo-red conjugated crude synaptic fractions by unstimulated or stimulated astrocytes (3 TSC and 3 control in technical duplicates from two separate experiments). **(D)** Western blot analysis of MEGF10 after 24-h cytokine stimulation conditions. **** indicates *p* < 0.0001, ***p < 0.0001, **p < 0.001, *p < 0.05. NT: not treated; A1: C1q, TNF-α, IL-1α treated; A2: TNF-α, IL-1β treated. Mann–Whitney *U* tests without multiple test correction were performed to determine significance. *SPARCL1* (WT vs. TSC; NT: Log2 FC –7.48, *p* < 0.0001; A1: –7.71 *p* < 0.0001; A2: –6.78, *p* = 0.0003), *GAS6* (WT vs. TSC; NT: Log2 FC –2.29, *p* = 0.0085; A1: –1.68, *p* = 0.0418; A2: –1.3, *p* = 0.1416), *MERKT* (WT vs. TSC; NT: Log2 FC –2.29, *p* = 0.1649; A1: –1.68, *p* = 0.3132; A2: –1.3, *p* = 0.122), *MEGF10* (WT vs. TSC; NT: FC log2 –3.75, *p* = 0.0003; A1: –2.07, *p* = 0.0005; A2: –1.9, *p* = 0.0012).

The ability to remove debris from the environment is an important process for maintaining a healthy neuronal environment and reducing inflammatory responses (Zhou et al., 2021). To investigate this capacity, we interrogated our model with qPCR on known phagocytic proteins in astrocytes. Here, we found mRNA downregulation of phagocytosis receptor *MEGF10*, downstream interactor *GAS6* and synaptosomal interacting protein *SPARCL1*. Whereas the receptor *MERTK* did not show any significant changes (Figure 3A). Conversely, we could not see a clear MEGF10 downregulation on protein levels (Figure 3D). Despite this, we further investigated phagocytosis using live cell imaging as a functional readout.

For the functional readout of phagocytosis, we incubated the (un)stimulated cell with pHrodo-conjugated crude synaptic fraction that emits a stronger fluorescent signal upon lysosomal incorporation. This process was tracked using live cell imaging and qualitatively assessed with a confocal microscope to show the overlap with post-incubation added lysosomal staining. Using this method, we observed a severe reduction of phagocytosis capabilities [WT vs. TSC; NT: fold change (FC) log2–1.32, *p* = 0.0043 A1: -2.08, *p* = 0.0043; A2: -2.18, *p* = 0.0043], that were not significantly altered by inflammation stimuli (Figure 3C). Since mTOR signaling is hyperactive in TSC astrocytes, we also investigated whether inhibition of mTOR by the small molecule rapamycin influenced the phagocytic response. Surprisingly, the uptake of particles remained impaired during mTOR inhibition (Figure 3C). With the use of confocal imaging and the lysosome staining we could also visually observe that the labeled particles were not incorporated into lysosomes, the amount and size of lysosomes were seemingly increased compared to control (Figure 3B and Supplementary Figures S2, S3 for condition overview).

Overall, phagocytic activity in TSC astrocytes is significantly downregulated, and this downregulation could not be rescued by mTOR inhibition or stimulated by inflammatory cytokines.

### Single-nuclei RNA sequencing reveals key pathway alterations in TSC astrocytes

**Figure 4:**
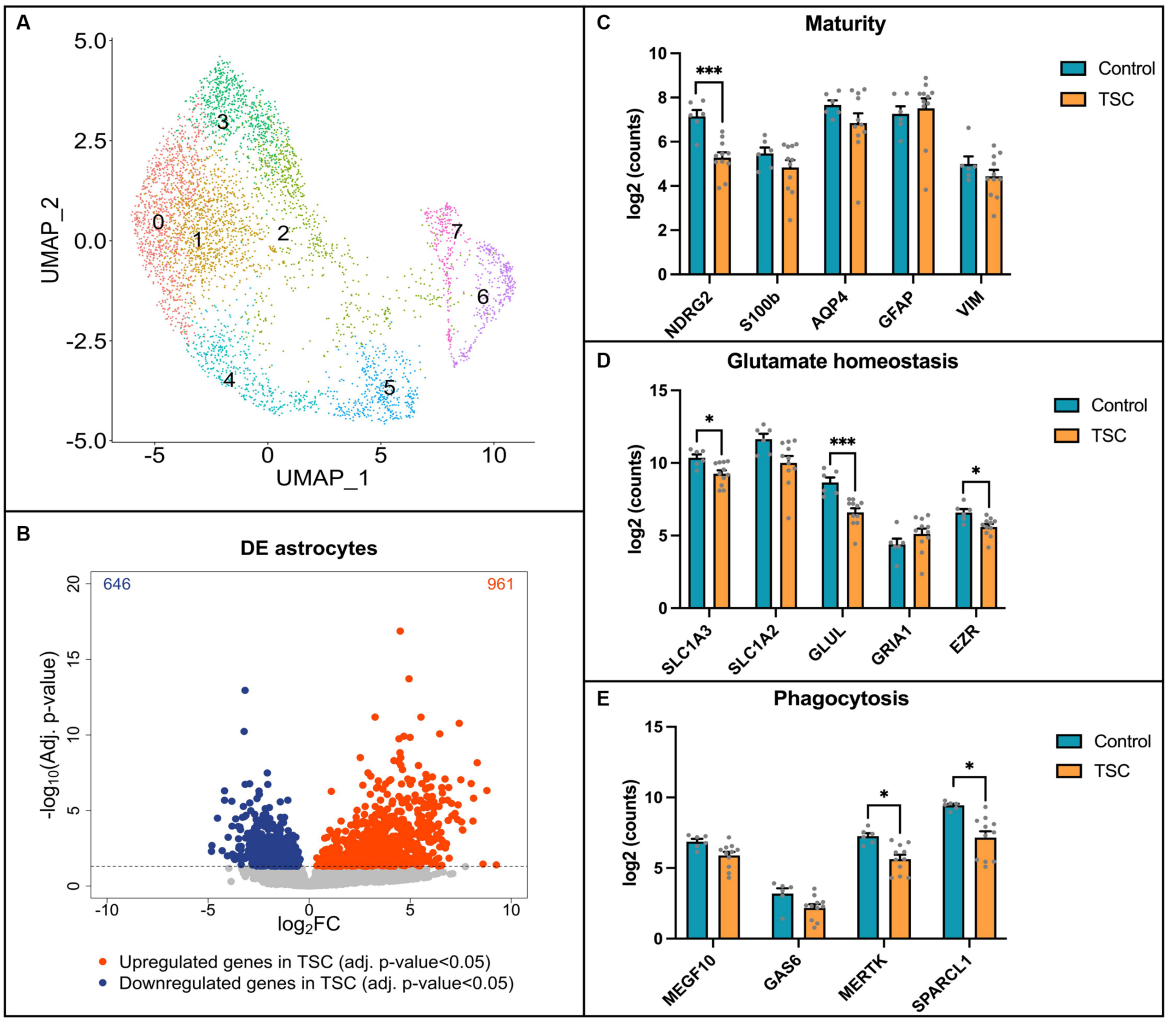
Single-nuclei RNA sequencing reveals alterations in key pathways in TSC astrocytes. **(A)** Uniform Manifold Approximation and Projection (UMAP) plot displaying the clustering of astrocytes. Eight distinct clusters were identified and found in both control (*n* = 6) and Tuberous Sclerosis Complex (TSC) (*n* = 11) tissue. **(B)** Volcano plot illustrating the differential gene expression analysis based on control (*n* = 6) and TSC (*n* = 11) samples. A total of 646 downregulated genes and 961 upregulated genes were identified in TSC when compared to control samples. The analysis was performed using DESeq2 after pseudobulk of the count values. Genes with a value of *p* less than 0.05 were considered statistically significant and are depicted in blue (downregulated) or red (upregulated) dots. **(C)** Box plot representation of log2-transformed count values for genes associated with astrocyte maturity. Notably, the expression of *NDRG2* was significantly downregulated in TSC compared to control samples. **(D)** Box plot depicting the expression of genes related to glutamate homeostasis. In TSC, there was a downregulation of *SLC1A3* (*EAAT1*), *GLUL* (*GS*), and *EZR* indicating alterations in glutamate regulation and metabolism. **(E)** Box plots showing the expression levels of genes involved in astrocyte phagocytosis. The expression of *MERTK* and *SPARCL1* was significantly downregulated in TSC, suggesting a dysregulation of astrocyte-mediated phagocytic processes. Statistical significance is denoted as * (*p* < 0.05) or *** (*p* < 0.001) in all box plots as determined by DESeq2, indicating significant differences between control and TSC samples. Samples were corrected for multiple comparison (Benjamini–Hochberg).

Next, we performed single-nuclei RNA sequencing (snRNA-seq) on frontal cortex tissue obtained from 6 control individuals and 11 patients diagnosed with tuberous sclerosis complex (TSC) to validate our *in vitro* model (Supplementary Table S1). The tissue samples were dissociated, and single-nuclei suspensions were prepared for library preparation and sequencing. Astrocytes were isolated computationally based on *GFAP*, *ALDH1L1*, *AQP4*, *GLT1*, and *S100B* gene expression signatures (the complete snRNA-seq dataset will be published elsewhere). To visualize the heterogeneity of astrocytes across the samples, we employed Uniform Manifold Approximation and Projection (UMAP) analysis. The UMAP plot revealed eight distinct clusters within the astrocyte population (Figure 4A). Notably, none of these clusters were found to be specific to TSC samples, indicating a lack of clear astrocyte subpopulations associated with the disease in this cohort. To identify differentially expressed genes between control and TSC samples, we performed a differential expression analysis. The analysis was conducted using DESeq2, after pseudobulk aggregation of the count values. Our analysis revealed a greater number of upregulated genes compared to downregulated genes in the TSC samples (Figure 4B, 646 vs. 961, *p* < 0.005). This suggests an overall increase in gene expression associated with the disease condition. The top 10 up- and downregulated genes are displayed in Supplementary Table S4. All differentially expressed genes were used to perform enrichment analysis and showed glial related processes such as glial cell differentiation (GO: 0010001), gliogenesis (GO: 0042063), and regulation of neurotransmitter levels (GO:0001505) (Supplementary Table S5).

To further compare our *in vitro* model to human brain tissue, we investigated the expression of specific genes in the snRNA-seq data. We focused on assessing the maturity of astrocytes, their ability for glutamate buffering, and their involvement in phagocytosis using the same set of genes investigated in our *in vitro* model. First, we assessed the maturity of astrocytes (Figure 4C) and observed a significant downregulation of NDRG2 in the TSC samples (log2FC: -2.119, *p* < 0.001), indicating a potential impairment in astrocyte maturation. Next, we investigated the expression of genes associated with glutamate buffering in astrocytes (Figure 4D). Our analysis revealed robust downregulation of EZR (log2FC: -1.109, *p* < 0.05), as well as glutamate transporter 1 (*SLC1A3/EAAT1*, log2FC: –1.151, *p* < 0.05) and the glutamate-ammonia ligase enzyme (*GLUL/GS*, log2FC: –2.346, *p* < 0.001), which are crucial factors in glutamate homeostasis (Mahmoud et al., 2019; Limbad et al., 2020). Furthermore, we explored the expression of genes related to astrocyte-mediated phagocytosis (Figure 4E). Interestingly, we identified downregulation of the phagocytic receptor *MERTK* (log2FC: –1.585, *p* < 0.05) and the synapse-associating protein *SPARCL1* (log2FC: –1.890, *p* < 0.05) in the TSC samples.

Overall, the snRNA-seq data shed light onto the astrocyte-specific alterations observed in our *in vitro* model, providing insights into the molecular changes associated with TSC. However, the observed variations in gene expression highlighted the need for further investigation and consideration of potential subpopulation-specific differences within astrocytes.

## Discussion

In this study, we utilized a combination of snRNA-seq together with a primary *in vitro* model to assess the changes observed in human TSC derived astrocytes on cell maturity and two homeostatic mechanisms; glutamate buffering and debris phagocytosis. Our results demonstrate that both glutamate and phagocytic capacity are impaired in TSC astrocytes in our *in vitro* model. Furthermore, a subset of our targets was also reflected in the snRNA-seq dataset. These findings offer evidence of systemic destabilization resulting from dysfunctional astrocytes, which, in turn, may contribute to the exacerbation of pathological hallmarks such as epilepsy and neuroinflammation.

### Maturity

In our study, we employed various markers that have previously been utilized to assess the maturity of astrocytes. These markers included: GFAP, S100B, VIM, AQP4 and NDRG2. While all of these markers were detected in our model system, some differences were observed compared to brain tissues, particularly in the case of GFAP. Reduced GFAP expression has been reported in other experiments using similar inflammation stimuli (Hyvärinen et al., 2019), suggesting these changes might be the result of mono-culturing or other technical causes. Additionally, we utilized S100B, a protein known to signal the transition of GFAP-positive cells to a mature stage of development (Raponi et al., 2007). We also examined vimentin, a well-known immature and reactive marker that has been described as an astrogliosis marker (Hol and Pekny, 2015) and associated with immature states (Luo et al., 2017). Our results indicate that TSC astrocytes exhibit reduced levels of GFAP and S100B but similar levels of vimentin compared to our controls, suggesting an immature state in TSC astrocytes. Finally, we used the maturity markers AQP4 and NDRG2. Of these markers, AQP4 is associated with the end feet and regulate water homeostasis with a close relation to the blood brain barrier, but can also modulate seizures (Verkman et al., 2006). Both blood brain barrier leakage and seizures are pathological hallmarks in TSC (van Vliet et al., 2015; Nguyen et al., 2019). NDRG2, a tumor suppressor, is typically present in non-reactive mature astrocytes, and it associates with neuronal nerve terminals, partly overlapping with AQP4 localization at these synapses (Flügge et al., 2014). The reduced association with synapses, resulting from the downregulation of NDRG2 and AQP4 observed in our TSC astrocytes, could potentially lead to a decrease in neuronal network stability.

### Glutamate buffering in astrocytes

Aberrations in glutamate metabolism have been reported in mouse models of TSC (Zeng et al., 2007), and the regulation of glutamate by astrocytes has been shown to mitigate seizures (Zeng et al., 2010). Furthermore, abnormal glutamate activity has reported in other brain pathologies such as TLE (Davis et al., 2015), general epilepsy (Çavuş et al., 2016) and neurodegeneration (Scimemi et al., 2013), suggesting a common underlying mechanism. The dysregulation of glutamate transporters (SLC1A3/EAAT1, SLC1A2/EAAT2) has been observed in resected human tissue (Boer et al., 2010), affecting both astrocytes (Sosunov et al., 2008) and abnormal giant cells (González-Martínez et al., 2011). Glutamate receptors exhibit deficiencies in human TSC tissue, closely correlating with mTORC1-induced S6 phosphorylation (Talos et al., 2008). Our results are consistent with these findings, as we observe robust downregulation of glutamate receptors, leading to elevated extracellular glutamate levels. Furthermore, our single-nucleus RNA sequencing (snRNA-seq) data partially overlaps with our *in vivo* observations of glutamate receptor downregulation. In addition to these downregulated receptors, EZR is required for motility of the perisynaptic astrocyte processes (Lavialle et al., 2011; Badia-Soteras et al., 2023), interaction of the astrocytic leaflets with the synaptic cleft (Badia-Soteras et al., 2023) and glutamate/ion homeostasis (Schacke et al., 2022). Furthermore, immune-reactive states (neurotoxic/protective) have also been correlated with a neuroprotective role of EZR (Payán-Gómez et al., 2018), which is related to complement and interferon activity – two elements overexpressed in TSC (Boer et al., 2010; Zhang et al., 2015). Overall, these downregulations could play a part in our observed dysregulation by reduction of glutamate intake and sensing.

Once imported, the glutamate will need to be converted to glutamine. Glutamine synthetase (GLUL/GS), an enzyme predominantly expressed by astrocytes, plays a central role in converting glutamate to glutamine in the human brain (Schousboe et al., 2014). However, this process can be influenced by environmental factors such as mitochondrial dysfunction (Ebrahimi-Fakhari et al., 2016) and inflammation (Boer et al., 2010; Zhang et al., 2015) both of which have been previously described in the context of TSC. In our experiments, GLUL was downregulated independent of inflammation in the *in vivo* system. Moreover, snRNA-seq has shown a downregulation of GLUL, providing additional evidence of this deficiency.

In our primary patient-derived cell model, we observed differences in the essential machinery between control and TSC that were reflective of their ability to control the extracellular glutamate. It is likely that the results presented in this study reflect the collective impact of various elements, including transporters and enzymes, which together contribute to the inability to regulate glutamate levels effectively.

### Phagocytosis in astrocytes

The phagocytic capability of astrocytes has recently been described in literature (Konishi et al., 2020). This process is primarily mediated through scavenging receptors like MEGF10 and MERTK (Morizawa et al., 2017), while microglia primarily employ TREM2 (Takahashi et al., 2005), CR3 (Hong et al., 2016) and P2Y6R (Xu et al., 2016). However, conflicting evidence exists regarding whether astrocytes or microglia predominate in phagocytosis. This likely depends on factors such as brain region, substrate type, and the functional status of microglia (Damisah et al., 2020; Konishi et al., 2020). In our study, we did not find alterations in MEGF10, MERTK or GAS6 that could fully explain the severe loss of phagocytosis in TSC astrocytes. It is likely that the mechanism behind impaired phagocytosis in TSC astrocytes is more complex than a simple reduction of phagocytosis-associated proteins. These mechanisms could include downstream signaling (Morizawa et al., 2017), metabolics (Baik et al., 2019), lysosomal machinery and/or proper uptake and subsequent processing. Recent evidence suggests that TSC dysfunction influences stress granules (Kosmas et al., 2021), including interactions between the TSC complex and structural lysosomal proteins LAMP1/2 through the adapter G3BP1 (Fitzian et al., 2021). Moreover, stress granule formation is altered by TSC1 and TSC2 deficiency (Fitzian et al., 2021), indicating a close interaction between TSC-related mechanisms and the lysosomal compartment. Surprisingly, our rapamycin inhibition did not improve the uptake of particles during the 24 h of exposure, suggesting insensitivity to mTOR signaling. Other targets that we examined such as SPARCL1 are not directly involved in phagocytosis, but perform an associative role with synapses and their formation. Their downregulation is known to cause impairment of synapse formation (Singh et al., 2016), plasticity (Jones et al., 2011) and has been associated with ER stress in autism spectrum disorder (Taketomi et al., 2022). Therefore, they could pose an interesting future target in respects to epileptic network formation in TSC.

In conclusion, dysfunctional cellular machinery may contribute to a heightened state of inflammation due to the inability to process debris, but the mechanisms underlying the reduced phagocytosis require further investigation.

### Future outlook

Here, we have demonstrated the dysregulation of key homeostatic functions in astrocytes isolated from TSC patients. A remaining question is whether these dysregulations can be partially resolved through therapeutic interventions. Indeed, certain chemicals have been used to stimulate phagocytosis in astrocytes (Morizawa et al., 2017) and some of these small molecules are currently undergoing clinical trials for other diseases (Mudaliar et al., 2013; Traussnigg et al., 2021). Furthermore, the mTOR inhibitor rapamycin has been described as having the ability to reduce glutamate levels by increasing the expression of glutamate transporters (Zeng et al., 2008). However, we have observed a decrease in phagocytic capabilities following the administration of rapamycin in our model. This observation is consistent with a previous study on macrophages (Fox et al., 2007). The downregulation could potentially interfere with the mechanisms involved in cell clearance, thereby exacerbating certain inflammatory processes. Ideally, a non-inflammatory phagocytic activator could be utilized to enhance the clearance of cellular debris, mitigating potential inflammatory processes. Furthermore, such activators might represent a promising dual-therapy strategy when combined with clinically available mTOR inhibitors.

### Technical limitations

A primary limitation of this study is the use of a mono-culture of astrocytes, which may oversimplify the complex environmental inputs and behaviors found *in vivo*, even when artificial stimuli like cytokines are used. For instance, microglia have been shown to greatly influence the behavior of astrocytes (Liddelow et al., 2017), as well as other cell types (Brambilla et al., 2014; Xanthos and Sandkühler, 2014; Jeong et al., 2021) and environmental cues (Castro et al., 2009). However, we attempted to address this limitation by utilizing a defined FCS-free media prior to our assays, aiming to better simulate the normal brain environment, particularly its resting and mature phenotype. These formulations were based on previous studies with astrocyte differentiation and non-reactive maturation (Shaltouki et al., 2013; Perriot et al., 2018; Bradley et al., 2019). Furthermore, our functional assays used physiological brain media formulation BrainPhys (Bardy et al., 2015) to better reflect the neural environment.

Another limitation pertains to the use of NeuN+ FACS sorting prior to snRNA sequencing, as the dataset was initially generated for different research purposes. Consequently, only 20% of the final nuclei consisted of glial cells, resulting in a lower number of astrocytes. Possibly, with an increase of astrocyte nuclei in the data, more significance could be achieved. It is worth noting that while the expression of other maturity-related genes showed no differences, the overall distributions of their expression exhibited larger variations. This variability may be attributed to the relatively low number of astrocytes across the TSC samples, making it challenging to further sub-cluster the data and identify potential subpopulation-specific differences.

## Data availability statement

The data analyzed in this study was obtained from Biotech Research & Innovation Center (BRIC), University of Copenhagen, Denmark, the following licenses/restrictions apply: the third-party producer of the data will not allow the data to be released to the public domain until 3 years post data generation. Requests to access these datasets should be directed to e.aronica@amnterdamumc.nl.

## Ethics statement

The studies involving humans were approved by medical research ethics committee (MREC) METC Amsterdam UMC. The studies were conducted in accordance with the local legislation and institutional requirements. Written informed consent for participation in this study was provided by the participants’ legal guardians/next of kin.

## Author contributions

ML: Conceptualization, Methodology, Validation, Visualization, Writing – original draft, Writing – review & editing. MS: Data curation, Formal analysis, Investigation, Writing – original draft, Writing – review & editing. FS: Data curation, Formal analysis, Investigation, Writing – review & editing. JA: Methodology, Writing – review & editing. WV: Resources, Writing – review & editing. IK: Methodology, Writing – review & editing. FJ: Resources, Writing – review & editing. KR: Resources, Writing – review & editing. PE: Resources, Writing – review & editing. PG: Resources, Writing – review & editing. JM: Data curation, Formal analysis, Supervision, Writing – review & editing. RK: Investigation, Writing – review & editing. TZ: Writing – review & editing. DB: Writing – review & editing. KK: Funding acquisition, Writing – review & editing. EA: Funding acquisition, Supervision, Writing – review & editing. AM: Funding acquisition, Supervision, Writing – original draft, Writing – review & editing.
