# Supplementary material for: Loss of maturity and homeostatic functions in Tuberous Sclerosis Complex-derived astrocytes
Source: Front Cell Neurosci. 2023 Nov 28;17:1284394. doi: 10.3389/fncel.2023.1284394 (PMC10713821; doi:10.3389/fncel.2023.1284394)
Supplement: Supplementary file 6 [file Data_Sheet_1.DOCX]

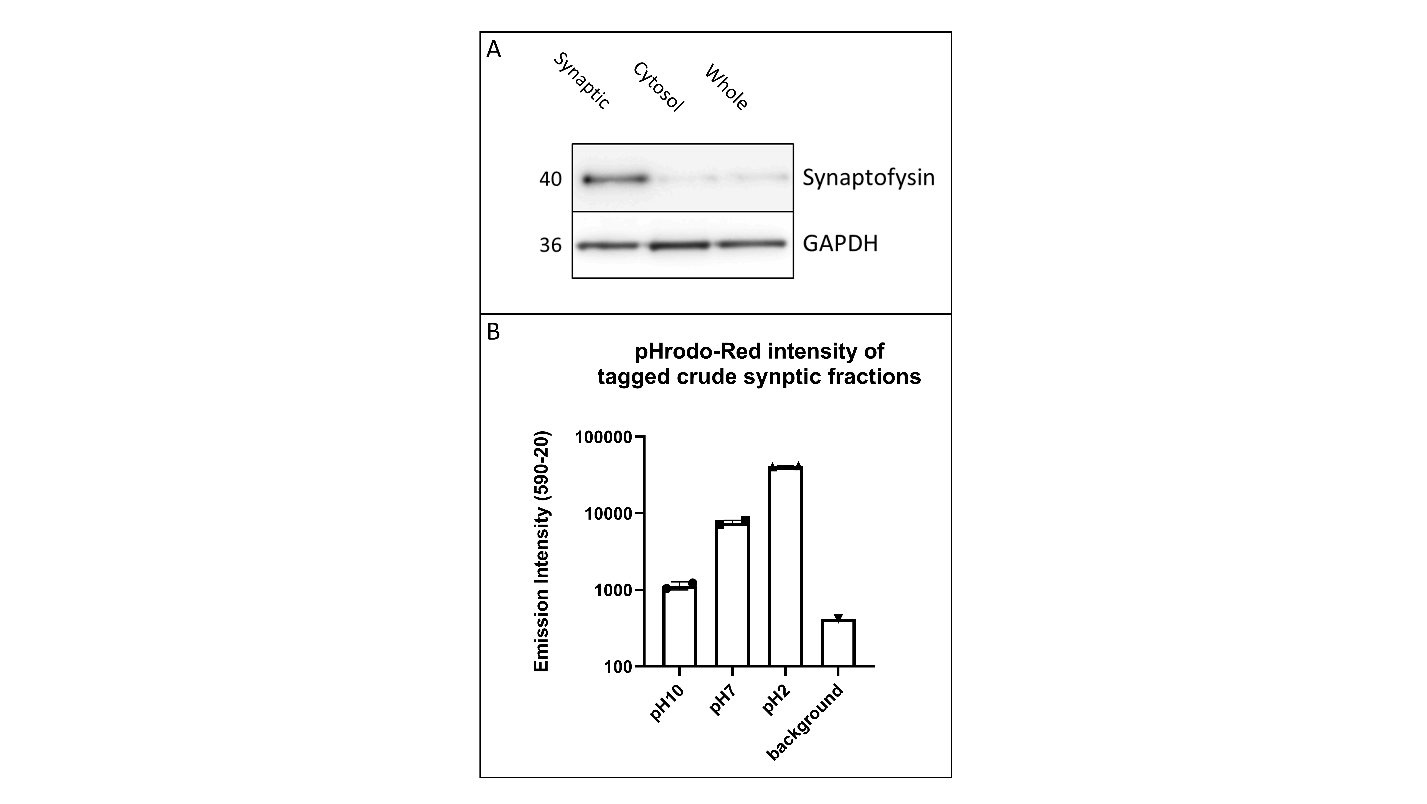

**Supplementary Figure 1**: Western Blot analysis of crude synaptosome fractions. **A:** Western blot of synaptophysin and GAPDH. **B:** pH titration of pHrodo tagged synaptic fraction measured in emission intensity.


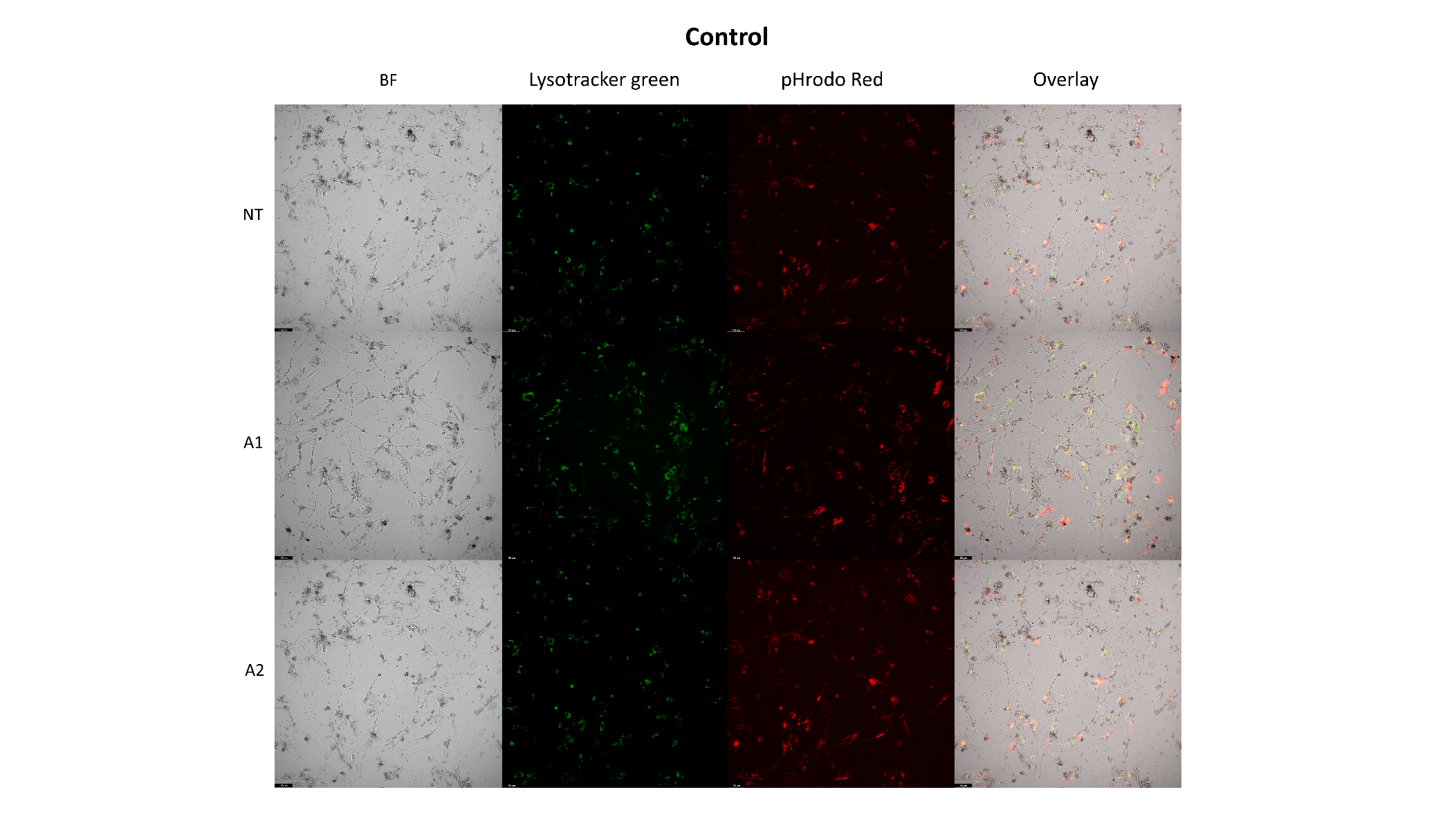


**Supplementary Figure 2:** 10x magnification images of 24-hour incubation with a pHrodo-tagged crude synaptosome fraction in non-treated (NT), TNF-α, IL-1α, C1q (A1) or TNF-α and IL-1β (A2) stimulation conditions – control line


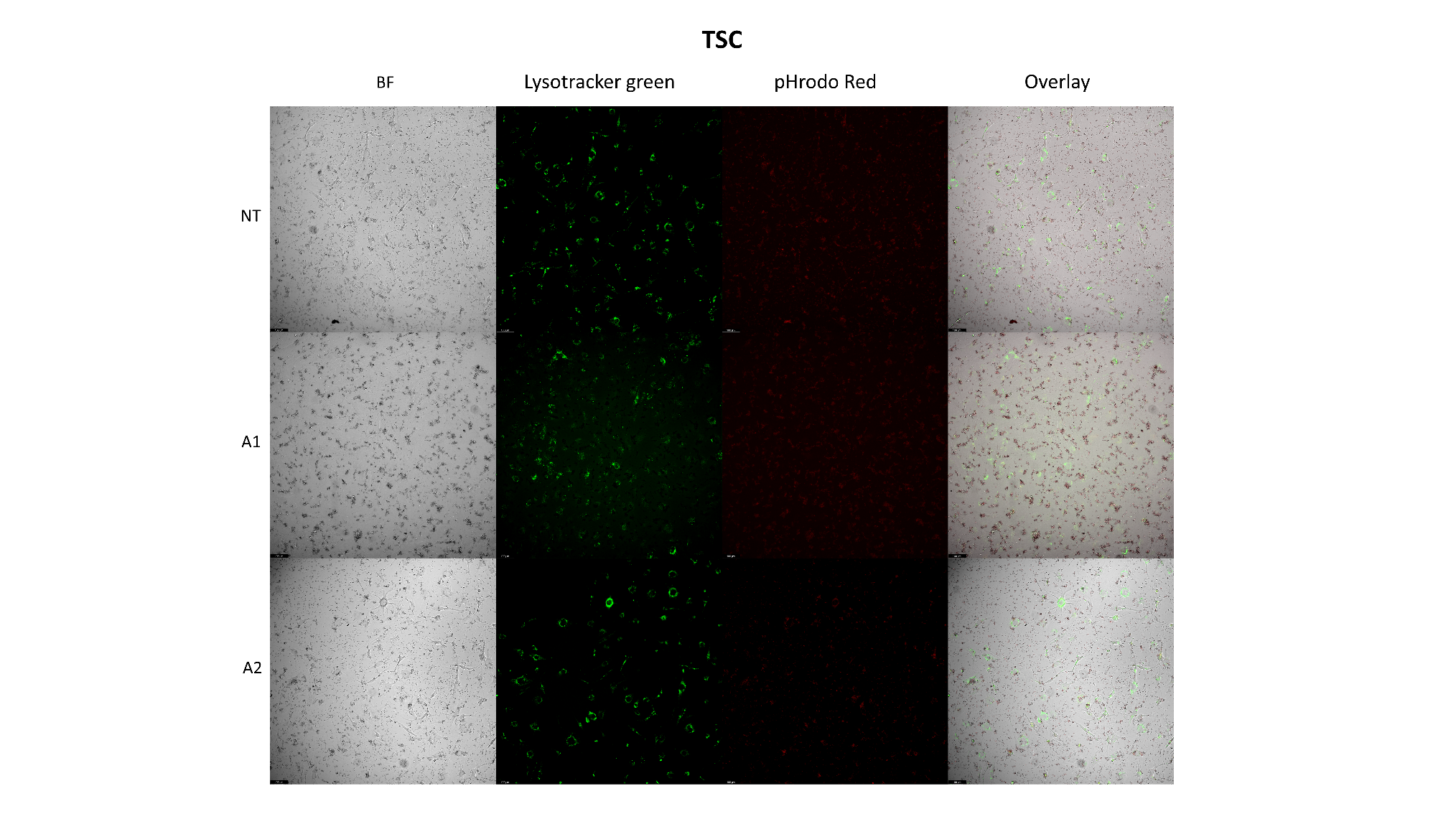


**Supplementary Figure 3:** 10x magnification images of 24-hour incubation with a pHrodo-tagged crude synaptosome fraction in non-treated (NT), TNF-α, IL-1α, C1q (A1) or TNF-α and IL-1β (A2) stimulation conditions – TSC line

**Supplementary methods**

Western Blot

Cells were lysed for 30’ at 4°C using radio immunoprecipitation assay buffer (RIPA) buffer (150 mM NaCl, 1% nonidet NP-40, 0.5% sodium deoxycholate, 0.1% sodium dodecyl sulfate (SDS), 50 mM Tris pH 7.4) supplemented with 2x Protease Inhibitor Cocktail Set Vl (Merck) and 1x PhosSTOP (Roche). Lysate was run through 25-gauge needle ~3-5 times to reduce viscosity and spun down 13,000 xg for 10’ at 4°C. The pellet was discarded and the supernatant was quantified using a BCA assay (ThermoFisher) in triplicate. For the SDS-Page protein input was used in combination with LDS loading buffer (NuPage) and DTT to a final concentration of 1x LDS and 100 mM DTT. Samples were heated for 10’ at 70°C, cooled and resolved on 17 well NuPAGE 4 to 12%, 1.0mm gels (Invitrogen) for 80’ at 100V volt with 1x MOPS (Bio-Rad) as running buffer and PageRuler Plus Prestained Protein Ladder (Invitrogen) as molecular size reference. Blotting was performed using a Mini Trans-Blot system with transfer buffer (25 mM Tris, 192 mM Glycine, 10% methanol) at 100V for 1h on ice. Post transfer the PVDF membranes were blocked with 5% bovine serum albumin (BSA) in Tris-buffered saline + 0.1% Tween-20 (TBST) for 1h at RT. For primary and secondary antibodies overview table; see supplementary table 2. All primary antibodies were incubated overnight at 4°C with agitation in antibody diluent (TBS, 5% BSA, 0.1% Tween-20). Membranes were washed 3 times with washing buffer (TBS, 0.1% Tween-20) and respective secondary HRP-conjugated antibodies (1:2500 for ECL or 1:12500 for ECL+) were incubated for 1h at RT. Afterwards, blots were washed 3 times. ECL solution (Pierce ECL Western Blotting Substrate, Thermofisher) or enhanced ECL solution (SuperSignal West Pico PLUS Chemiluminescent Substrate, Thermofisher) was used for development of membranes. The signal was captured with an ImageQuant LAS 4000 imager (GE Healthcare). When necessary, blots were stripped with Restore PLUS Western Blot Stripping Buffer (Thermofisher) according to the manufacturer’s instructions, blocked and reprobed.
